# Effects of statins on cytokines levels in gingival crevicular fluid and saliva and on clinical periodontal parameters of middle-aged and elderly patients with type 2 diabetes mellitus

**DOI:** 10.1371/journal.pone.0244806

**Published:** 2021-01-08

**Authors:** Huiyuan Zhang, Yameng Zhang, Xiaochun Chen, Juhong Li, Ziyang Zhang, Haiyang Yu

**Affiliations:** 1 State Key Laboratory of Oral Diseases & National Clinical Research Center for Oral Diseases & West China Hospital of Stomatology, Sichuan University, Chengdu, China; 2 Shenzhen Center for Chronic Disease Control, Shenzhen, China; University of the Pacific, UNITED STATES

## Abstract

**Objective:**

To analyze the effect of statins on cytokines levels in gingival crevicular fluid (GCF) and saliva and on clinical periodontal parameters of middle-aged and elderly patients with type 2 diabetes mellitus (T2DM).

**Methods:**

Systemically healthy controls (C group, n = 62), T2DM patients not taking statins (D group, n = 57) and T2DM patients taking statins (S group, n = 24) were recruited. In each group, subjects (40–85 years) were subclassified into the h (periodontal health)group, the g (gingivitis)group or the p (periodontitis) group according to different periodontal conditions. 17 cytokines in gingival crevicular fluid (GCF) and saliva samples of each subject were measured utilizing the Luminex technology kit. Further, HbA1c (glycated hemoglobin), FPG (fasting plasma glucose), PD (probing depth), CAL (clinical attachment level), BOP (bleeding on probing), GI (gingival index) and PI (periodontal index) were recorded. Data distribution was tested through the Shapiro-Wilk test, upon which the Kruskal-Wallis test was applied followed by Mann-Whitney U test and Bonferroni’s correction.

**Results:**

Levels of IFN-γ, IL-5, IL-10 and IL-13 in the saliva of the Dh group were significantly lower than those in the Ch group, while factor IL-4 was higher (p<0.05). Levels of MIP-3α, IL-7 and IL-2 in GCF of the Dh group were considerably higher than those in the Ch group (p<0.05), while that of IL-23 was considerably lower. Compared with the Cg group, levels of IFN-γ, IL-4, IL-5, IL-6, IL-10 and IL-13 were significantly lower in the saliva of the Dg group (p<0.05). Lower levels of IFN-γ, IL-5 and IL-10 were detected in the Sg group than those in the Cg group (p<0.05). At the same time, levels of IL-1β, IL-6, IL-7, IL-13, IL-17, IL-21 and MIP-3α in the gingival crevicular fluid of the Sg group were lower in comparison with the Dg group. In addition, lower levels of IL-4 and higher levels of IL-7 in GCF were identified in the Dg group than those in the Cg group, while in the Sg group, lower levels of IL-4, MIP-1αand MIP-3αwere observed than those in the Cg group (p<0.05). Lower levels of IFN-γ, IL-6, IL-10, IL-13 and I-TAC were found in the Sp group compared with those in the Cp group. The IFN-γ, IL-6 and IL-10 levels were lower in the Dp group than those in the Cp group (p<0.05). Meanwhile, in the Sp group, lower levels of pro-inflammatory factors IFN-γ, IL-1β, IL-2, IL-6, IL-7, IL-21 and TNF-α, in addition to higher levels of anti-inflammatory factors IL-4 and IL-5 in gingival crevicular fluid, were identified than those in the Dp group. Higher levels of IFN-γ,IL-1β,IL-2,IL-7,IL-21 and TNF-α and a lower level of IL-5 in the Dp group were identified than those in the Cp group (p<0.05). Moreover, statins were able to substantially reduce PD in T2DM patients with periodontitis, indicating an obvious influence on the levels of cytokines secreted by Th1 cells, Th2 cells and Th17 cells, as revealed by PCA (principal component analysis).

**Conclusion:**

Statins are associated with reduced PD and cytokines levels in the GCF and saliva of T2DM patients with periodontitis.

## Introduction

Periodontitis is a type of chronic, progressive, and infectious disease of periodontal supporting tissues predominantly caused by local irritating factors, such as plaque biofilms. Periodontitis has become one of the most common causes of tooth loss [1], being related to systemic diseases, including diabetes [2–4], cardiovascular diseases [5], hypertension [6], chronic kidney disease [7], chronic liver disease [8], peptic ulcers [9] and breast cancer [10].

Changes of periodontal local inflammatory factors are related to the progression of periodontitis [11], which is a result of plaque microbial infection and immune responses triggered by plaque biofilms. As the cytoderm component of Gram-negative bacteria in the plaque biofilm, Lipopolysaccharide (LPS) is able to activate mononuclear macrophages, thereby upregulating the secretion of interleukins (IL), macrophage inflammatory protein (MIP), colony stimulating factor (CSF), interferon (IFN), transforming growth factor (TGF) and tumor necrosis factor (TNF) [12]. Increased levels of pro-inflammatory factors, for instance, interleukins-1β (IL-1β), interleukins-6 (IL-6), tumor necrosis factor-α (TNF-α), and macrophage inflammatory protein (MIP-1α), as well as decreased levels of anti-inflammatory factors, namely interleukins-4 (IL-4), interleukins-5 (IL-5), and interleukins-10 (IL-10) are detectable in the local periodontitis lesions. These inflammatory factors synergistically interact with each other, thereby forming a complicated immune response that is critical in initiating and regulating host regulations of periodontal disease [13, 14]. Inflammatory factors render changes in periodontal tissues through different pathways. By illustration, pro-inflammatory factors can regulate the receptor activator of nuclear factor κB ligand (RANKL), which elevates the activity and number of osteoclasts and thus causes bone resorption [15, 16]. Fibroblast apoptosis is also induced by upregulating collagenolytic enzymes, prostaglandin E2 (PGE2) and chemokines [17]. Meanwhile, anti-inflammatory factors can downregulate pro-inflammatory factors and RANKL expression in activated T cells, thereby inhibiting osteoclastogenesis [18]. Type II diabetes mellitus (T2DM) is a type of metabolic disease caused by deficiency of insulin, where the pathogenesis thereof principally includes insufficient insulin production, insulin malfunction, and lack of insulin receptors on the cell surface, resulting in decreased glucose tolerance and increased blood sugar. A myriad of clinical and epidemiological studies have exhibited a two-way relationship between T2DM and periodontitis [4, 19]. The degree of periodontitis in T2DM patients is more serious than that in non-diabetic patients, particularly for those with poor blood sugar control and glycosylated hemoglobin. In parallel, increased levels of systemic pro-inflammatory mediators caused by periodontitis are not conducive to the blood sugar control of T2DM patients. Here, insulin resistance is aggravated, along with an increase in the risk of diabetes complications [2]. After periodontal treatment, FPG and HbA1c are majorly reduced in T2DM patients with periodontitis [20–22]. Dyslipidemia is one of the risk factors for the occurrence and progression of atherosclerosis in T2DM patients. The American College of Cardiology/American Heart Association cholesterol Guidelines (ACC/AHA) in 2013 recommended that T2DM patients must receive at least moderate statin treatment, this being primarily attributed to the fact that statins can lower the serum level of low-density lipoprotein (LDL). Moreover, statin treatment should continue even if the serum level of LDL is lower than 70 mg/dl, and the dosage of other statins should not be reduced. Said moderate statin treatment contributes to prevention against cardiovascular complications caused by diabetes, such as coronary heart disease and stroke [23]. Reports have articulated that, by stimulating bone regeneration and inhibiting osteoclast differentiation, non-surgical treatment in combination with topical application of statins can relieve periodontitis [24]. The reason for this is that statins can improve periodontal conditions by promoting bone regeneration and inhibiting osteoclast differentiation. Further, compared with only filling hydroxyapatite, a combination of simvastatin can substantially improve the bone regeneration of key mandibular defects in T2DM and osteoporosis rats by upregulating the vascular endothelial growth factor (VEGF) and bone morphogenic protein 2 (BMP-2) [25]. Simvastatin is able to abolish the upregulated MMP-1, which is involved in the destruction of periodontal tissues, in LPS and also the high glucose-induced gingival fibroblasts in vitro [26].

Principal Component Analysis (PCA) is a statistical method that applies dimensionality reduction in order to visualize data. Here, a group of potentially correlated variables can be transformed into a group of linearly uncorrelated variables through orthogonal transformation. Said group of variables after conversion is referred to as the principal component. In multiple studies, many factors related to a problem are often proposed for the purpose of analyzing the problem comprehensively. In this case, each variable reflects certain information related to the research in different levels. Feres et al. [27] adopted 40 kinds of oral microorganisms as the main component to distinguish aggressive periodontitis from periodontitis. Yet, no studies presently exist that have employed PCA to analyze the effects of statins on oral cytokines, especially with regard to the levels of cytokines secreted by different types of Th cells in patients with T2DM.

Prior research has demonstrated the role of statins in improving local periodontal inflammation. Despite this, the role of statins in the treatment of periodontitis and gingivitis of T2DM patients remains unclear. The aim of the present study was to assess the effect of statins on changing inflammatory factors in gingival crevicular fluid (GCF) and saliva and on clinical periodontal parameters of middle-aged and elderly patients with T2DM.

## Materials and methods

### Ethical review

Eligible subjects (n = 143) treated in the Department of Stomatology and Endocrinology, Shenzhen Center for Chronic Disease Control from December 2018 to May 2019 were recruited. Informed written consent to voluntarily participate was received from every participant and a clear explanation of the research was provided. Furthermore, the stipulations outlined by the World Medical Association’s Declaration of Helsinki and the CONSORT group were stringently followed throughout the course of conducting and reporting the present study. The present study was approved by the Ethics Committee of Shenzhen Center for Chronic Disease Control.

### Inclusion and exclusion criteria

Subjects who were within the age range of 40–85 years and had at least 20 teeth were eligible, and were classified into one of three groups: systemically healthy controls (C group, n = 62), T2DM patients not taking statins (D group, n = 57) or T2DM patients taking statins(S group, n = 24). In each group, patients were subclassified into the h group (periodontal health), the g group (gingivitis) or the p group (periodontitis) group according to the condition of periodontal diseases. The diagnostic criteria for periodontitis are premised on the 2017 WORLD WORKSHOP <Staging and grading of periodontitis: Framework and proposal of a new classification and case definition > [28]. A patient is a periodontitis case in the context of clinical care if: 1) Interdental CAL is detectable at ≥2 non-adjacent teeth, or 2) Buccal or oral CAL ≥3 mm with pocketing >3 mm is detectable at ≥2 teeth and the observed CAL cannot be ascribed to non-periodontal causes, such as: 1) gingival recession of traumatic origin; 2) dental caries extending in the cervical area of the tooth; 3) the presence of CAL on the distal aspect of a second molar and associated with malposition or extraction of a third molar, 4) an endodontic lesion draining through the marginal periodontium; and 5) the occurrence of a vertical root fracture. The diagnostic criteria for gingivitis consist of: 1) Free gums and gingival papillae have become bright red or dark red; 2) Edema of gingival tissue, thickening of gingival margin and hypertrophy of the gingival papilla; 3) Gingival tissue has become fragile with a lack of elasticity; and 4) Dental plaque or dental calculus on the tooth surface. Patients without the above symptoms or clinical signs were included in the periodontal health group, with the grouping and baseline characteristics being listed in Table 1. Exclusion criteria: (i) Subjects had other systematic diseases except for T2DM in the D and S groups (e.g. hypertension, hyperlipidemia, heart disease, rheumatoid arthritis, and tumor); (ii) Administration of antibiotics or periodontal scaling within 6 months; (iii) Taking medication long-term aside from statins and hypoglycemic drugs; (iv) Patients with aggressive periodontitis; (v) Pregnant and lactating women;(vi) smokers.

**Table 1:** Classification and mean ages of the study groups.

Systemic status	Periodontal status	Ages	Number
Systemically healthy controls (C)	Healthy (Ch)	60.14±9.91	22
	Gingivitis (Cg)	56.14±10.25	21
43 female, 20 male	Periodontitis (Cp)	55.79±15.76	19
T2DM patients (D)	Healthy (Dh)	60.67±8.60	15
32 female, 25 male	Gingivitis (Dg)	57.05±7.90	21
	Periodontitis (Dp)	61.00±8.45	21
T2DM patients with statins medication (SD)	Gingivitis (SDg)	59.67±7.10	12
7 female, 16 male	Periodontitis (SDp)	60.17±8.21	12
Total			143

### Collection of periodontitis and clinical paraments

Six sites of each tooth, except the third molar and the residual root covered by gingiva, were examined utilizing the Florida probe (USA), through which PD, CAL, BOP, GI and PI were recorded. Subjects were subclassified into the h group (periodontal health), the g group (gingivitis) or the p group (periodontitis) predicated on the probing results. In addition, BMI, FPG and HbA1c were recorded.

### Saliva collection

Smoking and alcohol consumption were forbidden one hour prior to saliva collection, and any lipstick was removed. At 9:00 in the morning, each subject was requested to rinse the mouth thereof. Thirty minutes later, saliva collection was performed under a non-stimulation condition in an effort to ensure repeatability. Each subject was asked to open the eyes thereof, sit quietly and slightly tilt the head thereof forward, while oral movement was required to be as minimal as possible. Saliva was collected using a saliva collection tube (SARSTEDT, Genman) in accordance with the instructions. Upon centrifugation for 2 minutes and 1,000*g, a clear saliva sample was yielded in the tube, which was preserved with the other samples at -80°C for use.

### GCF collection

The examined area was isolated by cotton rolls after cleaning any debris. Three Whatman paper filters (Whatman, England) (2×10 mm) were inserted into the gingival sulcus or periodontal pocket of four first molars, respectively and held for 30 seconds. If the first molar was missing, the second molar in the area was detected. Filters were then placed in a 1.5 mL tube containing 500 μL of PBS, and stored at -80°C for use.

### Analysis of inflammatory factors

TNF-α, Interferon-γ (IFN-γ), IL-1β, interleukins-2 (IL-2), IL-4, IL-5, IL-6, interleukins-7 (IL-7), interleukins-8 (IL-8), IL-10, interleukins-13 (IL-13), interleukins-17 (IL-17), interleukins-21 (IL-21), interleukins-23 (IL-23), MIP-1α, macrophage inflammatory protein-1β(MIP-1β) and macrophage inflammatory protein-3α(MIP-3α) in saliva and GCF were examined through the Luminex fluorescent technique, utilizing Milliplex Magentic Beads (Merck Millipore, BA, USA) in accordance with instructions from the manufacturer. The data obtained were analyzed with the Milliplex Analyst program (Merck Millipore, BA, USA).

### Statistical analysis

SPSS 26.0 was employed for statistical analysis, while data distribution was assessed by applying the Shapiro-Wilk test. The data did not follow a normal distribution, thus, non-parametric tests were applied. Differences observed in cytokines levels among the different groups were analyzed by either the non-parametric Mann-Whitney U test for binary variables(h group) or the respective Kruskal-Wallis test for those consisted of several independent groups(g group and p group). In the latter case, variations of statistical significance were further subjected to post hoc pairwise analysis by applying the Mann-Whitney U test and Bonferroni’s correction. PCA was conducted to evaluate the differences among the subgroups. Here, PCA was first performed on the levels of the above cytokines in saliva and gingival crevicular fluid. Subsequently, the cytokines were grouped pursuant to the correlation thereof with Th1, Th2 and Th17 premised on prior research. Further, the PCA was conducted with Th1-related, Th2-related and Th17-related inflammatory factors in saliva and GCF as factors.

## Results

### Influences of statins on metabolic parameters in T2DM patients

BMI, FPG and HbA1c were compared in subgroups of periodontal health, gingivitis and periodontitis. As can be observed in Table 2, no significant difference in BMI was detected between the Dh and the Ch group (*p*>0.05). BMI in the Sg group was similar to that in the Cg and Dg groups (*p*>0.05), but BMI in the Dg group was substantially higher than that in the Cg group (*p*<0.05). Significant differences in BMI were not observed among the Cp, Dp and Sp groups (*p*>0.05).

**Table 2:** Comparison of metabolic parameters with regard to the systemic status of patients in different periodontal conditions.

Subgroups	BMI		FPG		HbA1c	
**Ch**	22.60±2.25	p>0.05	6.70±1.33 A	*p<0.05	5.01±0.51 A	*p<0.05
**Dh**	23.79±2.42		9.75±2.86 B		7.21±1.78 B	
**Cg**	22.48±1.92 A	*p<0.05	6.20±0.80 A	*p<0.05	5.05±0.37 A	*p<0.05
**Dg**	26.06±4.22 B		9.70±2.41 B		7.10±1.13 B	
**Sg**	24.13±3.90 AB		8.99±3.36 B		6.57±.124 B	
**Cp**	23.61±2.71	p>0.05	6.14±0.92 A	*p<0.05	5.17±0.41 A	*p<0.05
**Dp**	24.18±2.78		8.51±2.46 B		6.79±1.28 B	
**Sp**	24.05±1.70		7.96±1.57 B		6.58±1.31 B	
**Total**	23.83±3.00		7.88±2.46		6.09±1.37	

*p*>0.05 indicated no significant difference between two groups;

*p<0.05 indicated a significant difference and A<B.

FPG was significantly higher in the Dh group than that in the Ch group (*p*>0.05). FPG in the Dg group was similar to that in the Sg group, with FPG of both groups being higher than that of the Cg group (*p*<0.05). In addition, FPG in the Dp group was similar to that in the Sp group, with FPG of both groups being higher than that in the Cp group (*p*<0.05).

In the periodontal health subgroups, the HbA1c level in the Dh group was substantially higher than that of the Ch group (*p*<0.05). Similar levels of HbA1c were detected in the Dg and Sg groups, which were higher than that in the Cg group (*p*<0.05). HbA1c was lower in the Cp group compared with that in the Dp and Sp groups (*p*<0.05), while the levels of the latter two groups were similar.

No major differences in BMI, FPG and HbA1c were observed among the periodontal health subgroups (Ch, Cg and Cp groups) (p>0.05). In T2DM patients not taking statins, major differences in BMI, FPG and HbA1c were not found among the Dh, Dg and Dp groups (p>0.05), which was also the case for T2DM patients taking statins (Sp and Sg groups) as well (p>0.05).

### Statins decrease PD in T2DM patients combined with periodontitis

PD and CAL were compared in subgroups of periodontal health, gingivitis and periodontitis. The PD, BOP, GI and PI values are the average of each value in the above sites in each patient’s mouth, while the CAL values are the maximum of the CAL values in the above sites in each patient’s mouth. As exhibited in Table 3, in subjects with healthy dental periphery and gingivitis, no significant differences in clinical parameters were identified (*p*>0.05). In the periodontitis group, PD in the Dp group was substantially higher than that in the Cp and Sp groups (*p<*0.05). No significant difference was found in the other clinical parameters among the Cp, Dp and Sp groups (*p*>0.05).

**Table 3:** Comparison of clinical parameters with regard to the systemic status of patients in different periodontal conditions.

Subgroups	PD(mm)		CAL(mm)		BOP(%)		GI		PI	
**Ch**	1.63 (1.49, 1.75)	p = 0.191	1.48 (1.30, 1.53)	p = 0.636	8.00 (3.50, 14.25)	p = 0.963	0.16 (0.07, 0.30)	p = 0.939	0.08 (0.04,0.12)	p = 0.867
**Dh**	1.69 (1.57, 1.83)		1.42 (1.24, 1.72)		9.00 (4.00, 11.00)		0.18 (0.08, 0.22)		0.08 (0.03, 0.16)	
**Cg**	1.83 (1.73, 1.98)	P = 0.059	1.56 (1.32, 1.78)	p = 0.260	13.00 (7.00, 18.50)	p = 0.308	0.26 (0.16, 0.40)	p = 0.277	0.24 (0.19, 0.32)	p = 0.419
**Dg**	1.93 (1.84 2.08)		1.68 (1.58, 1.81)		11.00 (5.50, 19.50)		0.22 (0.12, 0.41)		0.23 (0.18, 0.26)	
**Sg**	1.80 (1.73, 1.99)		1.63 (1.40, 1.84)		8.00 (2.50, 11.50)		0.18 (0.05, 0.23)		0.24 (0.19, 0.28)	
**Cp**	2.35 (2.06, 2.71)A	Cp-Dp	3.25 (2.86, 3.63)	p = 0.567	18.00 (14.00, 34.00)	p = 0.409	0.42 (0.30, 0.72)	p = 0.377	0.68 (0.42, 0.83)	p = 0.785
		*p = 0.044								
**Dp**	2.65 (2.38, 3.01)B	Dp-Sp	3.02 (2.68, 3.52)		14.00 (6.50, 25.00)		0.40 (0.15, 0.61)		0.65 (0.34, 0.87)	
		*p = 0.016								
**Sp**	2.24 (1.98, 2.59)A		3.23 (2.76, 3.93)		18.00 (7.00, 26.50)		0.36 (0.08, 0.53)		0.72 (0.60, 0.87)	
Total	1.93(1.74,2.26)		1.76(1.53,2.86)		12.00(6.00,19.00)		0.24(0.12,0.42)		0.24(0.16,0.59)	

*p*>0.05 indicated no significant difference between two groups;

*p<0.05 indicated a significant difference and A<B.

### Influences of statins on inflammatory factors in saliva and GCF

**Fig 1:**
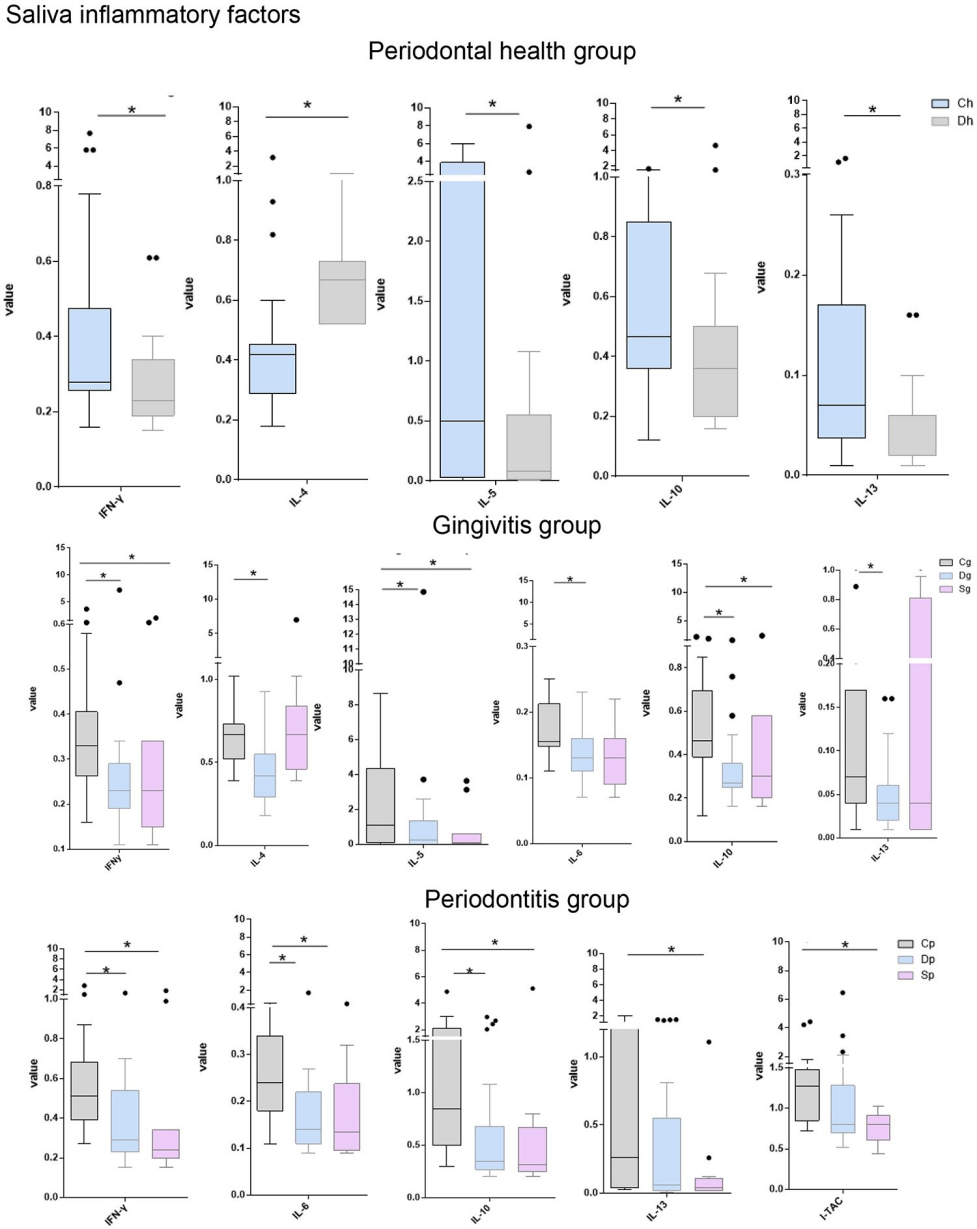
Cytokines levels in saliva and GCF, in pg/mL. Median values are represented by horizontal lines, interquartile ranges by boxes and outliers by points. Stars indicate a significant difference.

Levels of inflammatory factors in saliva are reported in Fig 1. In subjects with healthy dental periphery, levels of IFN-γ, IL-5, IL-10 and IL-13 in saliva in the Dh group were substantially lower than those in the Ch group, while factor IL-4 was higher (p<0.05). In subjects with gingivitis, compared with the Cg group, levels of inflammatory factors IFN-γ, IL-4, IL-5, IL-6, IL-10 and IL-13 were significantly lower in saliva in the Dg group (p<0.05). Lower levels of inflammatory factors IFN-γ, IL-5 and IL-10 were observed in the Sg group than those in the Cg group (p<0.05). In subjects with periodontitis, lower levels of IFN-γ, IL-6, IL-10, IL-13 and I-TAC were found in the Sp group compared with those in the Cp group. Moreover, the levels of IFN-γ, IL-6 and IL-10 were lower in the Dp group than those in the Cp group (p<0.05).

**Fig 2:**
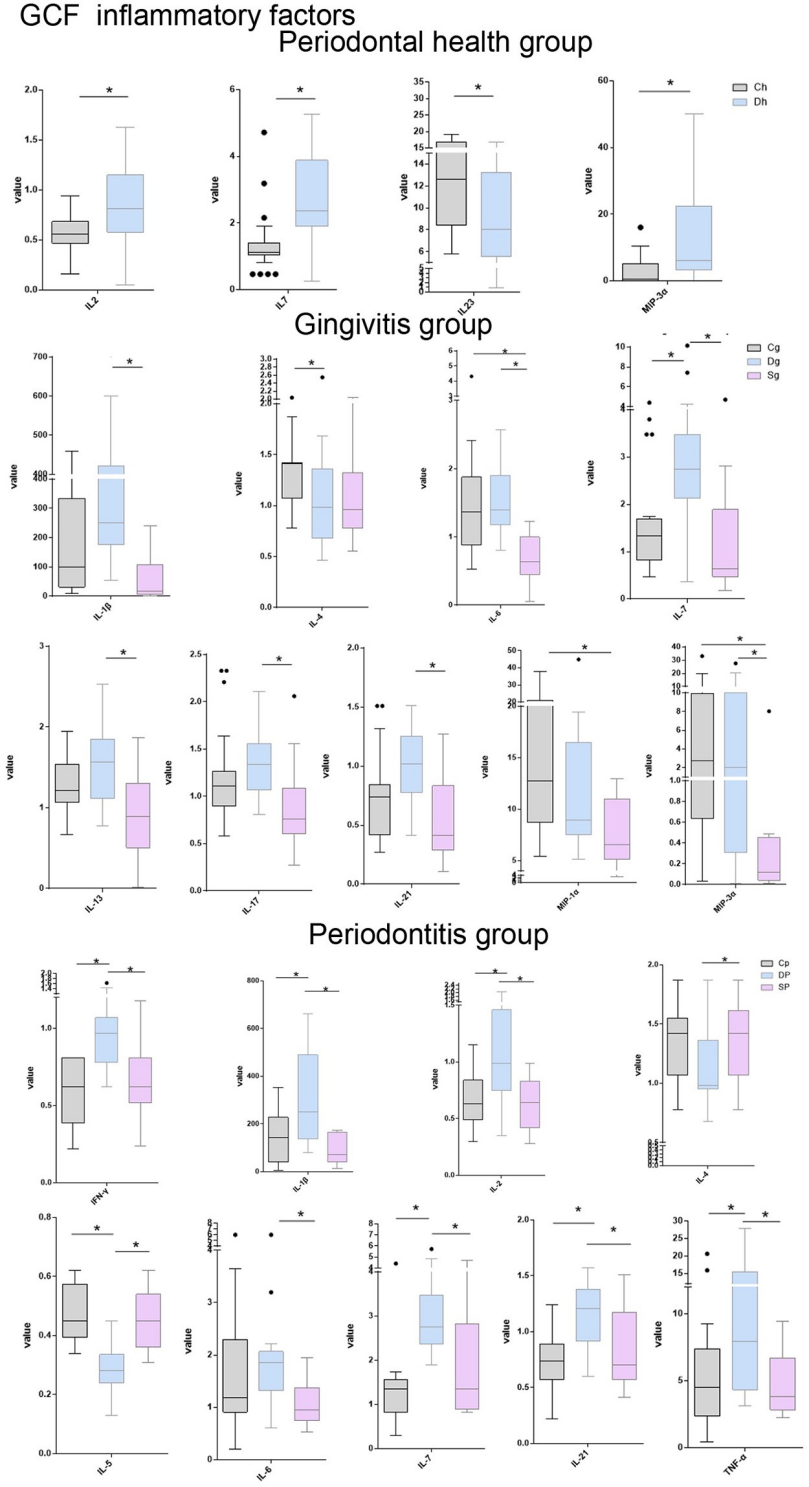
Cytokines levels in saliva and Saliva, in pg/mL. Median values are represented by horizontal lines, interquartile ranges by boxes and outliers by points. Stars indicate a significant difference.

Levels of inflammatory factors in GCF are presented in Fig 2. The levels of inflammatory factors MIP-3α, IL-7 and IL-2 in the gingival crevicular fluid in the Dh group were substantially higher than those of the Ch group (p<0.05), while the levek of IL-23 was considerably lower. In patients with gingivitis, levels of IL-1β, IL-6, IL-7, IL-13, IL-17, IL-21 and MIP-3α in gingival crevicular fluid in the Sg group were lower in comparison with the Dg group. Lower levels of IL-4 and higher levels of IL-7 in gingival crevicular fluid were detected in the Dg group than those in the Cg group. Meanwhile, in the Sg group, we lower levels of IL-4, MIP-1αand MIP-3αwere found than those of the Cg group (p<0.05). In patients with periodontitis, lower levels of pro-inflammatory factors IFN-γ, IL-1β, IL-2, IL-6, IL-7, IL-21, TNF-αand higher levels of anti-inflammatory factors IL-4 and IL-5 in gingival crevicular fluid in the Sp group were identified than those in the Dp group. Higher inflammatory factors IFN-γ, IL-1β, IL-2, IL-7, IL-21 and TNF-α and lower inflammatory factors IL-5 in the Dp group were observed than those in the Cp group (p<0.05).

### PCA of inflammatory parameters

**Fig 3:**
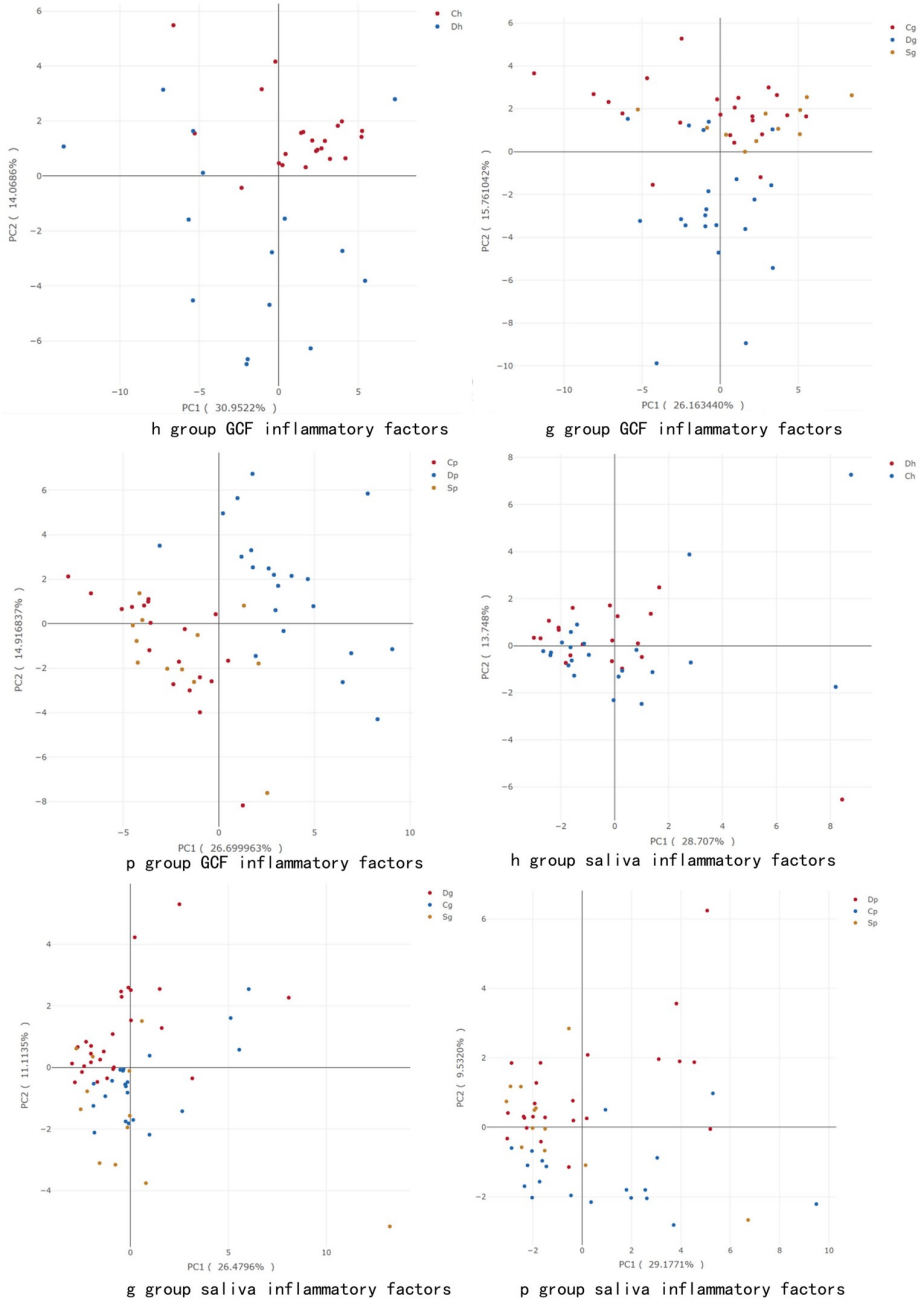
Principal component analysis according to baseline characteristics and cytokines in GCF and saliva.

PCA was conducted in consideration of inflammatory factors in saliva (TNF-α, IFN-γ, IL-1β, IL-2, IL-4, IL-5, IL-6, IL-7, IL-8, IL-10, IL-13, IL-17, IL-21, IL-23, MIP-1α, MIP-1β, MIP-3α, MPO and HCRP) and GCF (TNF-α, IFN-γ, IL-1β, IL-2, IL-4, IL-5, IL-6, IL-7, IL-8, IL-10, IL-13, IL-17, IL-21, IL-23, MIP-1α, MIP-1β, MIP-3α) of the subjects. The results are illustrated in Fig 3. In the periodontal health group, the C and D groups could be distinguished predicated on inflammatory factors. In gingivitis and periodontitis groups, the S and D groups could be also be distinguished based on inflammatory factors, while the differences between the S and C groups were minimal. Further, the distribution difference was more obvious in GCF than saliva. In the figure, PC1 and PC2 denote the contribution rates of the two principal components that had the largest impact on the grouping after the principal components were obtained through dimensionality reduction analysis. An observation could be made that the larger the sum of PC1 and PC2, the greater the contribution of these influence factors on the final grouping result. The sum of PC1 and PC2 in Fig 3 were less than 50%, highlighting that further grouping of inflammatory factors is needed before PCA analysis.

### Influences of statins on Th1, Th2 and Th17-related inflammatory factors in subjects

**Fig 4:**
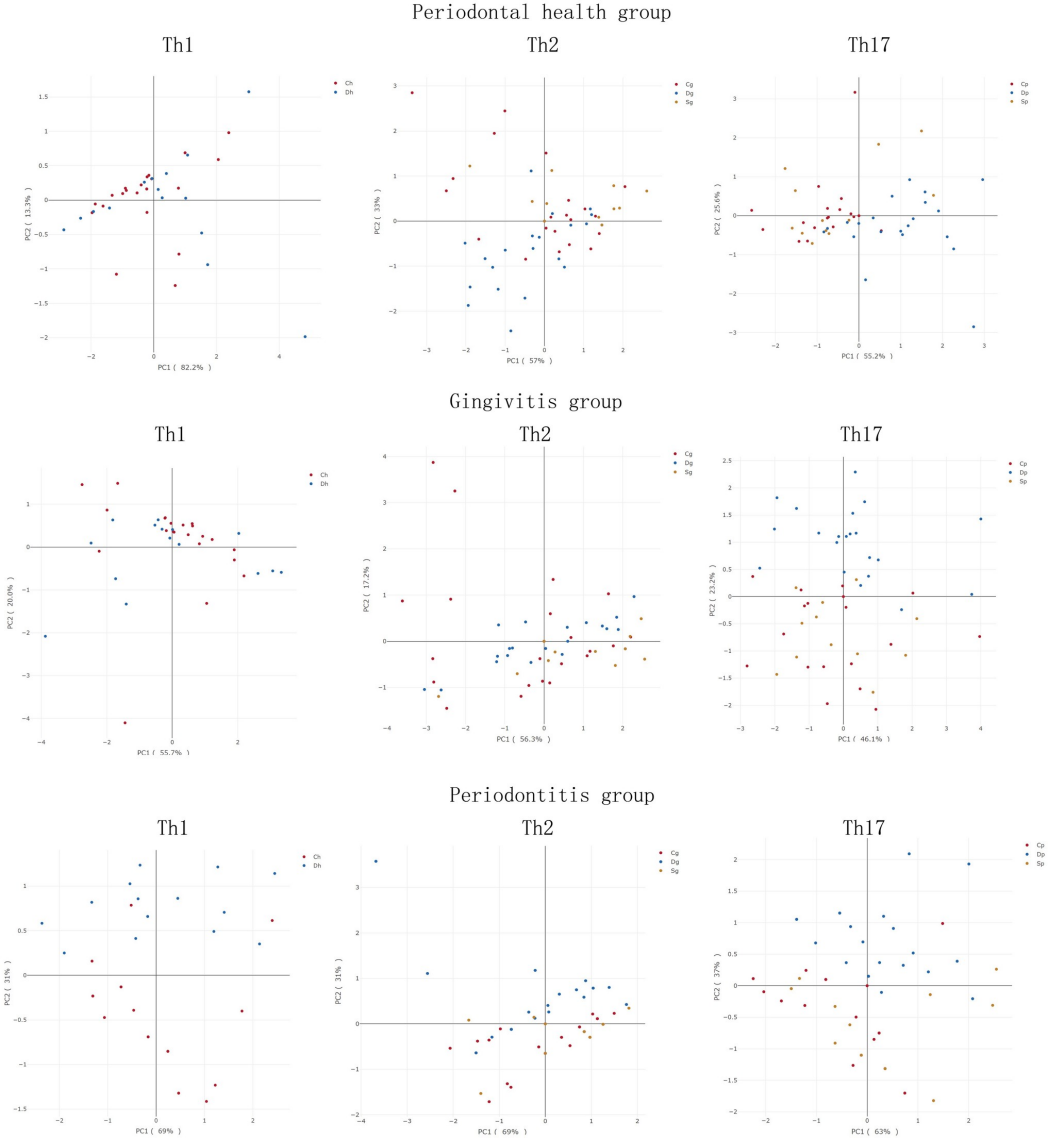
Principal component analysis premised on Th1, Th2 and Th17-related inflammatory factors.

On the basis of the produced cytokines, inflammatory factors were classified into one of three groups of Th1 (IL-2, IL-12 and IFN-γ), Th2 (IL-4, IL-5, IL-6, IL-10 and IL-13) and Th17 (IL-23 and IL-17). Differences between the subgroups were compared by depicting PCA images. As revealed in Fig 4, the sum of PC1 and PC2 was greater than 65%, implying that this grouping method is significant for principal component analysis of inflammatory factor levels. Additionally, clustering was obvious between the Dh and Ch groups, although the differences thereof in cytokines secreted by Th1, Th2 and Th17 cells were not obvious. An obvious clustering was observed among the Cg, Dg and Sg groups, while the differences in cytokines secreted by Th1 and Th2 cells were not significantly different. However, IL-17 and IL-23 secreted by Th17 cells were clustered in the Sg and Cg groups, and their differences were much more pronounced compared with those of the Dg group. Cytokines secreted by Th1, Th2 and Th17 cells were clustered in the Sp and Cp groups, and the differences were obvious in comparison with those of the Dp group. The above demonstrates that in the periodontitis group, statins had an obvious effect on the levels of Th1, Th2 and Th17 related cytokines in patients with T2DM. At the same time, in the gingivitis group, statins had an obvious effect on the Th17 related cytokines in patients with T2DM. A reasonable assumption can be made that statins may affect the metabolism and secretion of Th17 cells in patients with gingivitis and Th1, Th2 and Th17 cells in patients with periodontitis, thereby changing the level of cytokines and the state of periodontal tissue.

## Discussion

The present study assessed the effect of statins on inflammatory factors and periodontal paraments in saliva and GCF of healthy subjects and T2DM patients with different conditions of periodontal health. In general, compared with T2DM patients not taking statins, the differences in the levels of inflammatory factors in saliva of T2DM patients taking statins were less significant than those in gingival crevicular fluid. Statins exhibited a more obvious effect on downregulating inflammatory factors in T2DM patients with periodontitis than those with gingivitis, especially those in GCF compared with saliva. Compared with systemically healthy subjects with healthy dental periphery, the levels of IL-7, IL-2 and MIP-3α were higher, while the level of IL-23 was lower in GCF of T2DM patients. From the present understanding, there is a scarcity of research on inflammatory factors in GCF of T2DM patients with gingivitis. The present study uncovered that T2DM increased the level of IL-7 in GCF of patients with gingivitis, while the IL-4 level was decreased. IL-1β, IL-6, IL-7, IL-13, IL-17, IL-21 and MIP-3α in GCF of T2DM patients with gingivitis were significantly downregulated by statins treatment. Overall, the systemic inflammatory state of T2DM had an effect on the local inflammation in periodontal tissues. Existing studies have compared the expression differences in TNF-α, IL-6, IFN-γ, IL-17 and IL-23 in GCF of T2DM patients and systemically healthy subjects with periodontitis, which were higher in the former group [29–34]. Several studies have revealed that compared with systemically healthy subjects with periodontitis, T2DM patients combined with periodontitis had a higher level of IL-1β in GCF, while other cytokines were similar between them [30]. In patients with periodontitis, the present study observed increased levels of pro-inflammatory factors IFN-γ, IL-1β, IL-2, IL-7, IL-21, and TNF-α, in addition to decreased levels of anti-inflammatory factors IL-5 in GCF of T2DM patients. Owing to a high glucose state, enhanced oxidative stress in the local periodontal tissues triggers the production of advanced glycation end products (AGEs), which leads to increased levels of proinflammatory factors and decreased levels of anti-inflammatory factors by interacting with special receptors on gingival cells [35].

Statins exhibit pleiotropic effects, being able to improve vascular endothelial function, anti-inflammatory, antioxidant, immunomodulatory and antibacterial effects [36–40]. For this reason, statins have been extensively applied in T2DM treatment. The present results highlight that in T2DM patients systemically treated with statins, levels of pro-inflammatory factors IL-1β, IL-6 and TNF-α were elevated in GCF, whilst the anti-inflammatory factor IL-10 was downregulated compared with T2DM patients not taking statins. Consistent with the research on metabolic syndrome and systemically healthy population, systematic use of statins improves clinical periodontal parameters, as well as systematic or local inflammatory factors [26, 41–45]. As found in the present study, in T2DM patients with periodontitis, statins treatment reduced pro-inflammatory factors IFN-γ, IL-1β, IL-2, IL-6, IL-7, IL-21 and TNF-α levels in GCF, and enhanced anti-inflammatory factors IL-4 and IL-5 levels through the anti-inflammation and anti-oxidation effects [46]. Both anti-inflammatory and pro-inflammatory factors form a complicated immune network [13]. Despite this, clinical research has drawn an inconsistent conclusion thereon because of differences in systematic health state, local stimulation on periodontal tissue and other confounding factors. Up to now, evidence supporting the role of statins on inflammatory response in GCF of T2DM patients requires further analysis, particularly with regard to T2DM patients with gingivitis.

In the present study, systematic use of statins significantly reduced PD in T2DM patients with periodontitis, implying that statins possess an improving effect on periodontal parameters. This is consistent with a previous study on systemically healthy subjects [26]. Statins exert a protective role in the alveolar bone by inhibiting osteoclast differentiation via downregulating MMPs and RANKL [47, 48]. Further, statins are able to induce differentiation of periodontal ligament stem cells [49], and activate positive expressions of osteogenic markers in alveolar osteoblasts (AOB) and periodontal ligament cells (PDLC) in vitro [50]. However, experimental data has also revealed that statins are capable of inducing apoptosis of periodontal ligament fibroblasts and reducing cell viability [51, 52]. Conclusions on the protective effect of statins on periodontal tissues remain inconsistent, and the specific mechanism should be analyzed as well.

According to the cytokine production pattern of CD4+ T cells (T helper cells), the cells are divided into Th1, Th2, Th17, and Treg cells [13, 53, 54]. Th1 secretes IL-2, IL-12 and IFN-γ, while Th2 produces IL-4, IL-5, IL-6, IL-10 and IL-13 [13, 54]. Said cells all produce TNF-α and granulocyte-macrophage colony stimulating factor (GM-CSF). No significant differences in cytokines secreted by Th1, Th2 and Th17 were detected in subgroups of periodontal healthy people, highlighting that T2DM did not affect local inflammatory factors in periodontal healthy people since Th1, Th2 and Th17 signaling pathways were not affected by T2DM status in periodontal health tissue. In the gingivitis group, statins exhibited a huge impact on Th17-secreted cytokines, revealing that gingivitis activated Th17. IL-23 can induce Th17 cell differentiation, thus producing IL-17 [55–57]. Although studies have observed changes in the immune response of Th17 cells and changes in Th17-related cytokines like IL-17 and IL-23 at sites of periodontitis and peri-implant inflammation [57–59], no research has been conducted on Th17 cell-related factors in gingivitis patients as of yet. The limitation of the present study is that only the changes of cytokines were observed, without identifying the number and activity of Th17 cells. Hence, whether Th17 cells are initially altered prior to periodontitis developed from gingivitis should be further explored. Statins majorly affected Th1, Th2 and Th17-related cytokines in the periodontitis group, possibly being ascribed to LPS-activated mononuclear macrophages under the pathological state of periodontal infection and subsequent inflammatory response caused by the produced IL-1, IL-6 and TNF-α. Reports have indicated that statins inhibit myeloid DC-induced Th2 activation, and downregulate IL-4, IL-5 and IL-13 produced by Th2 cells [58]. Simvastatin improves experimental autoimmune encephalomyelitis by inhibiting the proliferation of pathogenic Th1 and Th17 cells [59]. The effect of statins on periodontal tissues, especially the secretion of inflammatory factors by Th1, Th2, and Th17 cells in diabetic models, and the mechanism thereof require further study.

GCF is a type of exudate and saliva is secreted by the salivary glands in the mouth. Differences in inflammatory factor levels in saliva were generally not as significant as those in GCF between T2DM patients treated with statins or not, implying that statins presented a larger effect on inhibiting inflammation in GCF. An assumption was made that oral administration of statins exerts the pharmacological function thereof after being metabolized by the liver and then into the circulatory system, while GCF mainly originates from the serum.

As depicted in the 2D-PCA images, the distribution difference was more obvious in GCF than saliva. In the gingivitis and periodontitis groups, the S and D groups could be distinguished, while differences between the S and C groups were small. This suggested that, similar to systematic healthy people, statins could downregulate the inflammatory response in T2DM patients with periodontitis. Meanwhile, clustering results were correlated to gender and age of T2DM patients. PCA provided the possibility to use the above-mentioned inflammatory factors in saliva and GCF as biomarkers to differentially diagnose gingivitis and periodontitis.

## Conclusions

Statins medication improves PD in T2DM patients with periodontitis, and influences local inflammatory response in T2DM patients with gingivitis and periodontitis, for the latter group in particular. Oral administration of statins is able to protect periodontal tissues in T2DM patients. However, as the present study is cross-sectional, the relationship between statins and the levels of cytokines was difficult to identify. Hence, the present results could be verified through further prospective experiments with more subjects.

## Supporting information

S1 File (ZIP)

S2 File GCF-1220. (SAV)

S3 File GCF-A-28SK.CUB. (XLS)

S4 File GCF-BC-28SK.CUB. (XLS)

S5 File Saliva-1220. (SAV)

S6 File SalivaAC-28SK.CUB. (XLS)

S7 File SalivaB-28SK.CUB. (XLS)

S8 File ZHY-28SK-0531.CUB. (XLS)
